# Gastric cancer is associated with *NOS2 *-954G/C polymorphism and environmental factors in a Brazilian population

**DOI:** 10.1186/1471-230X-10-64

**Published:** 2010-06-17

**Authors:** Yvana C Jorge, Marcia C Duarte, Ana E Silva

**Affiliations:** 1 UNESP, São Paulo State University, Department of Biology, Campus São José do Rio Preto, SP, Brazil

## Abstract

**Background:**

Gastric cancer can progress from a chronic inflammation of the gastric mucosa resulting from *Helicobacter pylori *infection that activates the inflammatory response of the host. Therefore, polymorphisms in genes involved in the inflammatory response, such as inducible nitric oxide synthase (*NOS2*), have been implicated in gastric carcinogenesis. The aim of this study was to evaluate the association of *NOS2 *polymorphisms Ser^608^Leu (rs2297518) in exon 16, -954G/C and -1173C/T, both in the promoter region, with gastric cancer and chronic gastritis and the association of cancer with risk factors such as smoking, alcohol intake and *H. pylori *infection.

**Methods:**

We conducted a population-based case-control study in 474 Southeast Brazilian individuals (150 with gastric cancer, 160 with chronic gastritis, and 164 healthy individuals), in which we performed *NOS2 *genotyping by PCR-RFLP.

**Results:**

SNP Ser^608^Leu was not associated with risk of chronic gastritis or gastric cancer. The polymorphic allele -1173T was not found in the studied population. However, the frequency of -954GC+CC genotypes was significantly higher (p < 0.01) in the cancer group (48.7%) than in both the gastritis (28.1%) and the control (29.9%) groups. Multivariate logistic regression showed that the *NOS2 *SNP -954G/C was associated with higher risk of gastric cancer (OR = 1.87; 95% CI = 1.12-3.13). We also observed an association with risk factors such as smoking and alcohol intake in both the gastric cancer (OR = 2.68; 95% CI = 1.58-4.53; OR = 3.60; 95% CI = 2.05-6.32, respectively) and the chronic gastritis (OR = 1.93; 95% CI = 1.19-3.13; OR = 2.79; 95% CI = 1.55-5.02, respectively) groups. This is the first report of increased risk of gastric cancer in association with the -954G/C polymorphism. These findings show that several polymorphisms in the promoter region of the *NOS2 *gene may contribute to the susceptibility to gastric cancer.

**Conclusions:**

Polymorphism *NOS2 *-954 G/C, along with alcohol intake and tobacco smoking, is associated with gastric cancer. However, the *NOS2 *Ser^608^Leu polymorphism was not associated with gastric carcinogenesis. The *NOS2 *-1173C/T polymorphism was absent in the studied population.

## Background

Gastric cancer, the fourth most common cancer in the world [1], is characterized as a multifactorial disease that results from individual genetic predisposition and exposure to environmental factors. Among the latter, the most strongly associated are *Helicobacter pylori *infection, diet, smoking and drinking [2]. In Brazil, gastric cancer ranges fourth in incidence and mortality, and an estimate of about 21,500 new cases was made for 2010 [3].

The *H. pylori *bacterium is the most important etiological factor related to chronic gastritis, which is a preneoplastic lesion, and gastric cancer [4,5]. Furthermore, it is present in 90% of all patients with chronic gastritis affecting the antrum [6]. *H. pylori*-induced gastric cancer occur through multi-step process that develops from active gastritis, to gastric atrophy, intestinal metaplasia, dysplasia, and finally to carcinoma [7]. This process consists of three major steps, characterized as *H. pylori *infection, gastric atrophy development, and carcinogenesis, which in each step, genetic traits interacting with lifestyle may influence the process [8]. Moreover it is assumed that several polymorphisms are involved in those steps, such as polymorphisms influencing gastric acid inhibition and innate immune response attenuation in *H. pylori *infection step; polymorphisms pertaining to the signal transduction in the step of gastric atrophy, and yet other polymorphisms related to final step of gastric carcinogenesis [8].

The inflammatory response to infection by *H. pylori *is an oxidative process originated by phagocytic cells that can lead to gastric injury [9]. This process is triggered by bacterial lipopolysaccharides, cytokines and products from the bacterial wall, inducing nitric oxide (NO) synthesis and resulting in reactive nitrogen species [10]. The main role of the NO production by host defense cells is to cause damage to the pathogen DNA, so it has antibacterial, antiviral and antitumoral properties [11].

NO is produced by the iNOS (inducible nitric oxide synthase) enzyme that is expressed by macrophages and neutrophils in gastric mucosa as part of the host response to the *H. pylori *infection, therefore increasing the production of NO, which has been associated to gastric carcinogenesis [12]. It has been speculated that increased levels of NO can damage host cell DNA, cause tissue injuries [13] and increase cell proliferation [14]. In addition, increased iNOS expression has been observed in patients with chronic inflammatory diseases [15], ulcer and gastritis [12], and solid tumors [16].

The iNOS enzyme is encoded by gene *NOS2*, and several SNP's (Single Nucleotide Polymorphisms) were described in it, such as the C/T substitution in exon 16 (rs2297518), which results in an amino acid substitution (Ser^608^Leu) [17], and -954G/C and -1173C/T, both in the promoter region of the gene that is linked to increased enzyme expression, resulting in higher NO production [18-23].

The Ser^608^Leu polymorphism was studied in a Spanish population [24], in reflux esophagitis, Barrett's esophagus, and esophageal adenocarcinoma in Caucasian subjects [25], and in prostatic cancer in non-Hispanic Caucasians and African-Americans [26].

Only one study reported the Ser^608^Leu polymorphism to be associated with gastric cancer among subjects with a history of smoking and alcohol intake [17], while polymorphisms -954G/C and -1173C/T have been associated with inflammatory processes such as malaria in populations of Tanzania [18], Gabon [19], Kenya [20] and Ghana [23], and osteomyelitis in Spanish subjects [27].

To our best knowledge, however, polymorphisms -954G/C and -1173C/T have not been studied in gastric cancer, and no allele or genotype frequencies are established for the Brazilian population for any of the referred SNPs. It is well accepted that both polymorphisms in the *NOS2 *promoter region cause overproduction of NO, suggesting that they might be observed in other diseases in which high NO levels are demonstrated, like in gastric carcinogenesis.

Inflammation has been recognized as a critical component in cancer progression. Several cancers arise from infection sites, chronic irritation and inflammation [28,29]. It is therefore reasonable to speculate that the genes involved in inflammatory host response to *H. pylori *infection could be relevant candidate genes to be studied in gastric cancer susceptibility. Thus, the aim of this study was to evaluate whether *NOS2 *polymorphisms Ser^608^Leu in exon 16 and -954G/C and -1173C/T, both in the promoter region, are associated with risk of chronic gastritis and gastric cancer in a Brazilian population and whether there is an association of cancer with risk factors such as smoking, alcohol intake and *H. pylori *infection.

## Methods

### Subjects

This was a case-control study on chronic gastritis and gastric cancer, in which a total of 474 DNA samples from peripheral blood leukocytes were genotyped. The case groups comprised 160 individuals (78 men and 82 women) with a histopathologically confirmed diagnosis of chronic gastritis - CG - (Sidney System) [30], with a mean age of 53 years (range 21-86 years), and 150 individuals (114 men and 36 women) with a histopathologically confirmed diagnosis of gastric adenocarcinoma - GA - (Lauren's classification) [31] with a mean age of 61 years (range 28-93 years). All subjects were recruited from the Hospital de Base in São José do Rio Preto, SP, and from the Pio XII Foundation in Barretos, SP, Brazil. *H. pylori *infection was histologically established by the Giemsa staining technique or by the urease test, performed at the Pathology Services of the Hospital de Base and the Pio XII Foundation. Results were obtained for the available cases. The cancer-free control group (C) with no previous history of gastric disease was composed of 164 healthy individuals (86 men and 78 women), mainly blood donors, with a mean age of 52 years (range 20-93 years). Epidemiological data on the study population were collected using a standard interviewer-administered questionnaire, with questions about current and past occupation, smoking habits, alcohol intake and family history of cancer. The National Research Ethics Committee approved this work, and written informed consent was obtained from all individuals.

### Genotyping

About 5 mL of whole blood were collected from all study participants in sterile EDTA-coated vacutainers. DNA was extracted according to a previous report [32], and stored at -20°C until use for genotyping.

The PCR-RFLP (Polymerase Chain Reaction-Restriction Fragment Length Polymorphism) technique was carried out to identify the *NOS2 *genotype according to the SNP features, which created a restriction enzyme recognition site, according to previously established protocols [17,18,33]. The PCR and DNA digestion conditions are summarized in Table 1. Briefly, PCR was performed in a total reaction volume of 25 μL, containing 2.5 μL 10× PCR buffer, 2 μL dNTPs (1.25 μmol/L), 0.5 μL MgCl_2 _(25 mmol/L), 1.25 μL of each primer (25 mmol/L, Sigma.), 15.3 μL dH_2_O, 2 μL DNA (100 ng/μL), and 0.2 μL Taq DNA polymerase (5 U/μL, Invitrogen). After an initial denaturation step at 94°C for 3 min, amplification was carried out by 35 cycles at 94°C for 30 s, at 61°C for 45 s, and at 72°C for 75 s, followed by a final elongation cycle at 72°C for 10 min. Then, 10 μL of PCR products were digested with 0.5 μL of the specific enzyme (range 5-10 U/°L) in a 10 μL volume including 2.5 μL 10× buffer 1 (NEB Corp.) and 7.0 μL dH_2_O. The products were then electrophoresed on a 2.5% agarose gel, to allow detection by ethidium bromide staining. In order to confirm the veracity of the results, one confirmed polymorphic case was used as positive control for every RFLP procedure, to attest the good functioning of the restriction enzyme. For the -1173C/T polymorphism, we also used another fragment of the *IL-1β *gene that we knew had the enzyme recognition site, to verify the correct functioning of the enzyme *BccI*, once we did not detect any heterozygous or polymorphic subjects.

**Table 1:** PCR-RFLP conditions for *NOS2 *gene polymorphisms and gene *IL-1β*.

Genes	Primers	Enzyme Time/T°	Fragments	Reference
***NOS2*** Ser^608^Leu rs2297518	F: 5' - TGTAAACCAACTTCCGTGGTG - 3' R: 5' - GTCTCTGCGGGTCTGAGAAG - 3'	*Tsp*509I (10 U/μL) 16 hs/65°C	288 bp CC:113, 175 bp CT:113, 142 and 175 bp TT:113, 142 bp	17
				
***NOS2*** **-954G/C**	F: 5' - CATATGTATGGGAATACTGTATTTCAG - 3' R: 5' - TCTGAACTAGTCACTTGAGG - 3'	*Bsa*I (5 U/μL) 1 h/37°C	573 bp GG:437, 136 bp GC: 573, 437 and 136 bp CC: 573 bp	18
				
***NOS2*** **-1173C/T**	F:5' - CAAAGATCCTTGAGCTCTGA - 3' R:5' - CAACTACATTAGGGAGAAGTTGAG - 3'	*BccI* (10 U/μL) 1 h/37°C	199 bp CC: 199 bp CT: 199, 132 bp TT: 132 bp	33
				
IL-1β	F:5' - CATGTGACCTGCTCGTCAGT - 3' R:5' - CCCTAGGGATTGAGTCCACA - 3'	*BccI* (10 U/μL) 1 h/37°C	372 bp 141 and 231 bp	-

T°, temperature.

### Statistical analysis

Fisher's exact test was used to compare the groups with regard to genotype and allele frequencies, and the chi-square test for determining Hardy-Weinberg equilibrium. Multiple logistic regression models were used to determine the effects of the variables in gastric cancer and chronic gastritis. The models included: age (reference: <57 years old - median of the groups), gender (reference: female), smoking habits (reference: nonsmokers), drinking habits (reference: nondrinkers), and *H. pylori *infection (reference: *H. pylori*-negative). The results are shown as odds ratios (OR) and their 95% confidence intervals (95% CI). OR's were calculated using a dominant model (i.e., combining heterozygotes and homozygotes for the minor allele) for all SNP's. Statistical analyses were performed using the GraphPad Instat and BioEstat computer software programs. A probability level (p) of less that 0.05 was adopted as significance criterion.

## Results

The genotype frequencies of the polymorphisms studied here were in Hardy-Weinberg equilibrium in both cases and control groups (data not shown). For Ser^608^Leu genotype frequencies, the comparisons between cancer and control groups (p = 0.81) and gastritis and controls (p = 0.72) did not show any statistically significant difference. Similarly, no significant difference was found between gastritis and control groups for -954G/C (p = 0.81), but there was a statistically significant difference between the cancer and the control group (p < 0.01), due to the higher frequencies of genotypes -954GC and -954CC in the gastric cancer group (Table 2). Genotypes -1173CT and -1173TT were absent in the studied population.

**Table 2:** Genotype and allele frequencies of *NOS2 *polymorphisms Ser^608^Leu, -954G/C and -1173C/T in gastric adenocarcinoma (GA), chronic gastritis (CG), and control (C) groups.

Genotypes/Alleles	GA N = 150 (%)	C N = 164 (%)	OR (95%CI)	p	CG N = 160 (%)	C N = 164 (%)	OR (95%CI)	p
Ser^608^Leu								
CC	106 (70.7)	113 (69)	0.92 (0.57-1.49)	0.81	107 (66.9)	113 (69)	1.10 (0.69-1.75)	0.72
CT	36 (24)	39 (23.8)			33 (20.6)	39 (23.8)		
TT	8 (5.3)	12 (7.2)			20 (12.5)	12 (7.2)		
**Alleles**								
C	0.83	0.81	0.88 (0.59-1.32)	0.61	0.77	0.81	1.24 (0.85-1.82)	0.29
T	0.17	0.19			0.33	0.19		
								
**-954G/C**								
GG	77 (51.3)	115 (70.1)	2.23 (1.4-3.53)	<0.01	115 (71.9)	115 (70.1)	0.92 (0.57-1.49)	0.81
GC	62 (41.3)	38 (23.2)			36 (22.5)	38 (23.2)		
CC	11 (7.4)	11 (6.7)			9 (5.6)	11 (6.7)		
**Alleles**								
G	0.72	0.82	1.74 (1.19-2.53)	<0.01	0.83	0.82	0.91 (0.6-1.36)	0.68
C	0.28	0.18			0.17	0.18		
								
**-1173C/T**								
CC	150 (100)	164 (100)	ND	ND	160 (100)	164	ND	ND
CT	0 (0)	0 (0)			0 (0)	0 (0)		
TT	0 (0)	0 (0)			0 (0)	0 (0)		
**Alleles**								
C	1	1	ND	ND	1	1	ND	ND
T	0	0			0	0		

N, Number of individuals; OR, Odds Ratio; CI, Confidence Interval; P, probability; ND, not done.

We also compared the genotype frequencies between the cancer and the gastritis groups (data not shown). No significance was found for Ser^608^Leu (p = 0.54), but for -954G/C there was a statistically significant difference between the groups (p < 0.01), due to higher frequencies of genotypes -954GC and -954CC in the gastric cancer group.

The potential associations between the distributions of *NOS2 *genotypes and exposure to risk factors for gastric cancer and chronic gastritis are presented in Table 3. In the gastric cancer group, the data show that male gender (OR = 1.79; 95% CI = 1.02-3.15; p = 0.04), age above 57 years (OR = 2.94; 95% CI = 1.77-4.90; p < 0.01), smoking habit (OR = 2.68; 95% CI = 1.58-4.53; p < 0.01), alcohol intake (OR = 3.60; 95% CI = 2.05-6.32; p < 0.01), and polymorphism -954G/C (OR = 1.87; 95% CI = 1.12-3.13; p = 0.01) were associated with a higher susceptibility to developing this neoplasm. The comparison between the gastritis and the control group showed that none of the polymorphisms were associated with chronic gastritis, and that male gender (OR = 0.52; 95% CI = 0.31-0.88; p = 0.01) had a protective role in the development of gastritis. Moreover, smoking habit (OR = 1.93; 95% CI = 1.19-3.13, p < 0.01) and alcohol intake (OR = 2.79; 95% CI = 1.55-5.02; p < 0.01) increased the risk for gastritis. For individuals tested for *H. pylori *infection (65 with gastric cancer and 125 with gastritis), statistical analysis showed that *H. pylori*-positive infection (OR = 3.20; 95% CI = 1.58-6.48, p < 0.01) increased the chance of developing gastritis (data not shown).

**Table 3:** Distribution of risk factors, genotypes of *NOS2 *polymorphisms Ser^608^Leu and -954G/C, and odds ratios (OR) for gastric adenocarcinoma (GA), chronic gastritis (CG), and control (C) groups.

Variables		GA × C			CG × C	
	**GA/C** **N**	**OR (95%CI)**	**p**	**CG/C** **N**	**OR (95%CI)**	**p**

**Gender**						
Female	36/78	Reference		82/78	Reference	
Male	114/86	1.79 (1.02-3.15)	0.04	78/86	0.52 (0.31-0.88)	0.01
**Age**						
<57 years	42/96	Reference		91/96	Reference	
≥57 years	108/68	2.94 (1.77-4.90)	<0.01	69/68	1.03 (0.64-1.65)	0.88
**Smoking**						
Nonsmokers	43/103	Reference		73/103	Reference	
Smokers	107/61	2.68 (1.58-4.53)	<0.01	87/61	1.93 (1.19-3.13)	<0.01
**Alcohol**						
Nondrinkers	65/132	Reference		100/132	Reference	
Drinkers	85/32	3.60 (2.05-6.32)	<0.01	60/32	2.79 (1.55-5.02)	<0.01
Ser^608^Leu						
CC	106/111	Reference		108/111	Reference	
CT+TT	44/53	1.19 (0.69-2.08)	0.51	52/53	1.12 (0.68-1.83)	0.64
**-954G/C**						
GG	77/112	Reference		116/112	Reference	
GC+CC	73/52	1.87 (1.12-3.13)	0.01	44/52	0.83 (0.50-1.38)	0.48

N, Number of individuals; OR, Odds Ratio; CI, Confidence Interval; P, probability.

## Discussion

*NOS2 *polymorphisms have been studied in several types of diseases, including malaria [18-20,23], diabetes [34], rheumatoid arthritis [35], osteomyelitis [36] and asthma [33], where NO production has been implicated in the disease pathogenesis. However, the role of *NOS2 *promoter region variants -954G/C and -1173C/T and of the Ser^608^Leu substitution in exon 16 in gastric carcinogenesis is still unclear. Therefore, in the present study, we investigated whether *NOS2 *polymorphisms affect the risk of developing gastric cancer and chronic gastritis in a Brazilian population. To our best knowledge, this is the first study that demonstrates an association of polymorphism -954G/C with risk of gastric cancer. Variant Ser^608^Leu was not associated with susceptibility to chronic gastritis and gastric cancer, and the polymorphic allele -1173T was not found in the population studied here.

Johannesen et al. [37] showed that the Ser^608^Leu polymorphism in exon 16 was one of the most frequent SNP's among 10 polymorphisms of the human *NOS2 *gene identified in a Danish population. These authors observed an association of this SNP with increased risk for a subset of type 1 diabetes in HLA DR3/4-positive individuals. The few studies that investigated the role of the Ser^608^Leu polymorphism in the risk of developing diseases related to an inflammatory process have shown conflicting results. For instance, Shen et al. [17] found a significant association between this polymorphism and the risk of gastric cancer only in patients with a history of smoking habit or alcohol intake. On the other hand, Canzian et al. [36] observed an association between the Ser^608^Leu polymorphism and atrophic gastritis, while Holla et al. [33] found this polymorphism to be associated with susceptibility to asthma.

The amino acid affected by this SNP is located in the catalytic domain of the protein, but the amino acid substitution is a conservative one, once it involves two amino acids with similar properties [36]. Canzian et al. [36] suggest that there is no selective pressure against either allele, and therefore the SNP does not result in a drastic change of function. However, the C>T substitution in the *NOS2 *gene may lead to a more activated iNOS in the target cells, finally elevating NO to a higher level [33].

In our study, we observed significantly higher frequencies of genotypes -954GC and -954CC in the gastric cancer group and also of risk factors such as smoking and alcohol intake compared to the healthy individuals. The relationship of drinking and smoking with iNOS activity has been reported previously. Alcohol consumption enhances neutrophil NO production *via *activation of iNOS, leading to neutrophil apoptosis [38]. In turn, ethanol oxidation generates acetaldehyde, which presents carcinogenic effects, since it interferes with many DNA synthesis and repair sites, leading to tumor development [39].

Smoking is a risk factor for gastric cancer because the chloroform and ethanol present in cigarettes act as cellular proliferation stimulators, besides increasing angiogenesis, VEGF (Vascular Endothelial Growth Factor) levels and the development of tumors. Furthermore, it has been shown that nicotine increases the activity of matrix metalloproteinases 2 and 9 and the protein expression of plasminogen activators such as urokinase-type plasminogen activator and its receptor, which are indicators of invasion and migration processes [40].

In the present study, *H. pylori *infection was observed in association only in the chronic gastritis group. The reduced number of individuals with an available *H. pylori *diagnosis in both the cancer and the gastritis groups may have led to some loss of statistical efficiency. Therefore, a larger sample is required to achieve greater statistical precision.

The -954C variant has a limited worldwide distribution [19], and its different frequency in mild versus severe malaria cases has led many authors to assume that the -954G/C mutation may play a role in innate immunity against malaria. Kun et al. [19] found higher frequencies of this polymorphism in malaria patients from Central Africa but not in German blood donors, and a lower frequency in samples from Southeast Asia (2%-4%). Similarly, Levesque et al. [18] observed an increased frequency of the same polymorphism in Tanzanian children with malaria (15%), while it was reduced in black Americans (6%) and absent in white Americans. In contrast, none of both polymorphisms, -954G/C and -1173C/T, was present in either a Papua New Guinea population with exposure to malaria [41] or in Czech asthma patients [33], which is in agreement with previous reports on Caucasian populations [18,27,36,41-43] where both polymorphisms were absent and seem to be ethnic-specific for the African population.

As for genotypes -1173CT and -1173TT, they were also absent in our study, but these polymorphisms are reported to be mainly prevalent in East African populations [43], as found in association with protection from malaria in Tanzanian and Kenyan children [20]. Boutilis et al. [41] observed higher expression of iNOS when either allele -954C or -1173T were present, and it was hypothesized that the substitution of -1173C/T could create a new sequence recognition site for the GATA-1 or GATA-2 transcription factors and consequently an increased degree of transcription. An alternative hypothesis would be that the -1173 C/T polymorphism disrupts the binding of a transcription repressor at this site [20].

It is important to highlight the fact that the three polymorphisms studied here increase iNOS activity, thus resulting in high NO production, which leads to the formation of reactive nitrogen species [10]. As a result, many oncogenic mutations may occur by mechanisms such as hOGG1 repair enzyme inhibition and inactivation of apoptosis through pro-apoptotic nitrosilation of proteins like p53 and caspases [44]. Moreover, an increased iNOS expression is related to the angiogenesis process [12].

When there are gastric injuries caused by *H. pylori *infection, the iNOS enzyme can play an important role in carcinogenesis, once the gastritis can persist for years and *NOS2 *stays active during all the process. Due to a positive feedback, the levels of NO increase up to a point that is highly toxic to the gastric cells, not only to the pathogen [44].

The Brazilian population displays great genetic heterogeneity as a consequence of high miscegenation during colonization, resulting mainly from the mating between natives Amerindians, African slaves (coming mostly from Africa's West Coast, such as Angola and the north of the Gulf of Guinea, and less from the counter-coast, such as Mozambique) and Europeans [45], which explains the differences from other studied populations. Gonçalves et al. [46] showed that most white Brazilians display high proportions of African and Amerindian mtDNA lineages, because of sex-biased genetic admixture with Europeans. So, the Brazilian population can have both African and European genetic components, which could explain the presence or absence of several polymorphisms in our study. Levesque et al. [18] also reported differences in *NOS2 *promoter polymorphisms and malaria severity between populations of Tanzania (East Africa), Gabon (Central Africa) and Gambia (West Africa), suggesting differences in the genetic background of these populations.

Thus, the distribution of genetic polymorphisms can vary among populations, depending on factors such as selective pressure, founder effect, migration, population sub-sampling and various socio-cultural factors that influence purely random mating [41]. Finding major differences among populations is therefore not surprising and indicates that the results of a study conducted in a given population should not be extrapolated to others. In addition, considering the large extension of Brazil, there may be differences in genotype distribution from one region to another due to different contributions of the three ethnic groups to the composition of the populations of these regions. Therefore, it is important that these findings are also investigated in other Brazilian populations.

## Conclusions

In conclusion, our study showed for the first time an association between the SNP in the *NOS2 *-954G/C promoter region and risk of gastric cancer, as well as an association with smoking and alcohol consumption in both gastric cancer and chronic gastritis in the Southeast Brazilian population evaluated. We, however, did not find any evidence of an association between the *NOS2 *polymorphism Ser^608^Leu in exon 16 and risk of gastric cancer or chronic gastritis, and did not detect the variant -1173T. Therefore, these results evidence that several *NOS2 *polymorphisms are associated with susceptibility to gastric cancer and emphasize the importance of such studies in populations of different ethnic extraction.

## Abbreviations

C: Control; CG: Chronic Gastritis; CI: Confidence Interval; dH_2_O: Distilled Water; dNTPs: Deoxyribonucleotide Triphosphates; EDTA: Ethylenediamine Tetraacetic Acid; GA: Gastric Adenocarcinoma; GATA-1: Erythroid Transcription Factor; GATA-2: GATA Binding Protein 2; hOGG1: Human 8-Hydroxyguanine DNA-Glycosylase; *IL-1β*: Interleukin 1-beta gene; iNOS: Inducible Nitric Oxide Synthase; MgCl_2_: Magnesium Chloride; mtDNA: Mitochondrial DNA; N: Number of Individuals; ND: Not Done; NO: Nitric Oxide; *NOS2*: Nitric oxide synthase 2 gene; OR: Odds Ratio; P: Probability; PCR-RFLP: Polymerase Chain Reaction - Restriction Fragment Length Polymorphism; SNP: Single Nucleotide Polymorphism; T°: Temperature; VEGF: Vascular Endothelial Growth Factor.

## Competing interests

The authors declare that they have no competing interests.

## Authors' contributions

YCJ carried out the molecular genetic studies, acquisition of data, performed the statistical analysis and drafted the manuscript. AES and MCD participated in the conception and design of the study, data interpretation, and contributed to the preparation and revision of the manuscript. All authors have read and approved the final manuscript.

## Pre-publication history

The pre-publication history for this paper can be accessed here:

http://www.biomedcentral.com/1471-230X/10/64/prepub
